# Impact of COVID-19 pandemic on HIV viremia: a single-center cohort study in northern Italy

**DOI:** 10.1186/s12981-021-00355-x

**Published:** 2021-06-04

**Authors:** Ilaria Izzo, Canio Carriero, Giulia Gardini, Benedetta Fumarola, Erika Chiari, Francesco Castelli, Eugenia Quiros-Roldan

**Affiliations:** 1 Division of Infectious and Tropical Diseases, ASST Spedali Civili, Brescia, Italy; 2 Department of Infectious Diseases, University of Brescia, Brescia, Italy

**Keywords:** HIV continuum of care, COVID-19, Public health, SARS-CoV-2, Follow-up, Adherence, HIV viremia

## Abstract

**Background:**

Brescia Province, northern Italy, was one of the worst epicenters of the COVID-19 pandemic. The division of infectious diseases of ASST (Azienda Socio Sanitaria Territoriale) Spedali Civili Hospital of Brescia had to face a great number of inpatients with severe COVID-19 infection and to ensure the continuum of care for almost 4000 outpatients with HIV infection actively followed by us. In a recent manuscript we described the impact of the pandemic on continuum of care in our HIV cohort expressed as number of missed visits, number of new HIV diagnosis, drop in ART (antiretroviral therapy) dispensation and number of hospitalized HIV patients due to SARS-CoV-2 infection. In this short communication, we completed the previous article with data of HIV plasmatic viremia of the same cohort before and during pandemic.

**Methods:**

We considered all HIV-patients in stable ART for at least 6 months and with at least 1 available HIV viremia in the time window March 01–November 30, 2019, and another group of HIV patients with the same two requisites but in different time windows of the COVID-19 period (March 01–May 31, 2020, and June 01–November 30, 2020). For patients with positive viremia (PV) during COVID-19 period, we reported also the values of viral load (VL) just before and after PV. Results: the percentage of patients with PV during COVID-19 period was lower than the previous year (2.8% vs 7%). Only 1% of our outpatients surely suffered from pandemic in term of loss of previous viral suppression.

**Conclusions:**

Our efforts to limit the impact of pandemic on our HIV outpatients were effective to ensure HIV continuum of care.

## Background

The impact of COVID-19 pandemic on the end of HIV epidemic by 2030, major aim of the last United Nations Programme on HIV/AIDS (UNAIDS), is of great concern around the world [1–4]. In fact, the pandemic has imposed dramatic challenges to the capacity of the global healthcare system [5–7].

Brescia Province, a high-density population area in Lombardy Region, northern Italy, was one of the worst COVID-19 epicenters in Europe during the first SARS-CoV-2 wave. The Infectious Division of ASST Spedali Civili Hospital of Brescia was highly involved from the very beginning in the fight against SARS-CoV-2 spread. As for other hospitals and health care facilities, human and economic resources were significantly converted to face the emergency. Therefore, health staff found itself facing two great challenges: ensuring the best medical management to inpatients with severe COVID-19 disease and guaranteeing a regular follow up to outpatients with chronic infectious diseases, such as the HIV-positive population, that counts for almost 4000 subjects in our outpatient Clinic.

On March 8, 2020 the Italian government imposed very restrictive community containment measures in order to limit the diffusion of SARS-Cov-2 (leaving home apart from health or working reasons demonstrated by a written self-declaration was strictly forbidden and health clinics had to guarantee social distancing measures). In addition, some of our patients were upset, feeling themselves more susceptible to the SARS-CoV-2 infection and its complications, then refusing to leave home even if a written self-declaration of health needing would have allowed it. From the very beginning (March 2020), we established an emergency procedure coordinated by an heterogeneous group of persons (medical doctors, nurses, pharmacists, IT technicians, consultants in sanitation) to modify the way the HIV health services for outpatients were provided, with the aim of both mitigating the effect of the pandemic on the “90–90–90” UNAIDS strategy and avoiding the exposure of HIV patients to the risk of acquiring the viral infection. We converted, when possible, outpatient in-person HIV consultations to telehealth visits and we assured the continuation of ART throughout home delivery services [8]. If a patient missed a (tele) visit, nursing or medical staff tried to trace him even in the next days, establishing a new appointment. Nurses and health assistants spent hours on the phone with patients every day, giving correct information about pandemic and related health risks, and reassuring patients, and counselling about the importance for them not to miss the medical evaluation and to assume regularly the ART. For patients unable to receive telematic visits (e.g. major comorbidities, difficulties in communication because of different spoken language or mental/neurological disorders, deaf and/or dumb people), we maintained face-to-face evaluation.

By the end of May 2020, the positive rate for SARS-CoV-2 infection decreased together with mobility restrictions (e.g. permission to practice sport, go to restaurants and bars, meet a limited number of friends and relatives, but respecting the inter-personal protection distance of at least 1 m and wearing the surgical mask) and patients realized that the risk of acquiring the infection were comparable to that of HIV-uninfected population. At that point, we had the time to re-organize our internal procedures to limit the unnecessary inter-personal contact and the number of face-to-face visits increased. In details, the time visit for each subject was prolonged to half an hour to ensure a limited number of patients in the waiting room, the visit room was disinfected and refreshed after each patient and a plastic glass on the table separated medical staff and visitors.

During the first 2 months of pandemic (March–April 2020) and despite our efforts, the number of missed follow up visits of HIV-patients raised from a mean of 5% during 2019 to 8% [8]. Moreover, an initial drop in ART drugs dispensation was observed (higher in March than April 2020 and then it returned to normal) and the number of newly diagnosed HIV patients reduced [8]. Our previous study [8] concentrated only on the impact of the pandemic on some indicators of the HIV care continuum, not considering the maintenance of the viral suppression and only referring to the period March–April 2020. However, during extraordinary situation like this, the relative burden of each indicator in understanding the individual-level retention in care may change [9]. Thereby, since the impact of pandemic and mobility restrictions on HIV care is still not clear, then we aimed to complete our previous research evaluating the pandemic effect on HIV viral load from March to November 2020 in our outpatient cohort.

## Methods

### Study population and data collection

The present retrospective observational cohort study was conducted at the University Department of Infectious and Tropical Diseases of the ASST Spedali Civili of Brescia, northern Italy.

We included HIV-infected patients who were on stable ART (defined as patients who assumed the same regimen of ART for at least 6 months) and had at least one HIV-RNA measurement between March 01-November 30, 2020 (COVID period). All ART regimens were chosen on the basis of genetic resistance test evaluation and pharmacological interactions. This cohort was exposed to more or less severe mobility and social distancing restrictions based on time period, as described in the previous section. Then, we distinguished between COVID period A (March 01–May 31, 2020) and COVID period B (June 01–November 30, 2020), because by the end of May 2020 these restrictions relieved, and we were able to guarantee a largest number of face-to-face visits, and the patient’s awareness of SARS-CoV-2 infection increased (see the previous section for details).

We also included all patients of our HIV cohort in stable ART for whom there was at least one viral load in the time window March 01–November 30, 2019. We defined this second population as the people who did not experience pandemic (pre-COVID period).

A PV was defined as HIV plasmatic VL > 50 copies/ml after 6 months of stable ART (unregarded ART lines). VL is usually measured every 5–6 months, unless PV is detected in the latest check or ART regimen changes leading to a closer check.

Patients with PV during COVID-19 period (period A or B) were considered only once, referring to the period of their first PV detection. For those patients, we also collected the values of the closest HIV viremia just before (5 or 6 months before PV) and after PV (1 or 3 months after PV). We identified as group 1 those patients with persistent PV; group 2 those who became PV during pandemic; group 3 those who had a single isolated PV during pandemic, and group 4 those for whom we had not yet further viremia test after PV.

Data were retrieved from electronic clinical charts. Since the majority of patients of the two cohorts overlapped, we used McNemar test on paired proportions for comparison of the two study periods. Results were described as absolute number of patients and percentages.

## Results

As of December 31, 2019, a total of 3875 HIV-infected patients were actively on follow up at our outpatient Clinic. From the beginning of March to end of November 2019, 3576 patients of our entire cohort were on stable ART from at least 6 months and had at least one plasmatic HIV-RNA available (92.3%). During this period, 254/3576 patients (7.1%) measured at least once a PV.

### COVID-19 period

During COVID period, 3537 patients were included in the study. Among them, 1322 patients during period A and 2215 patients during period B. Patients with PV during all COVID period were 99/3537 (2.8%) as follows, 73/1322 (5.5%) during COVID period A and 26/2215 (1.2%) during COVID period B.

Table 1 shows the number of patients with PV during COVID-19 period, divided by time window of first PV detection (period A vs period B) and by the closest values of HIV viremia just before and after PV.

**Table 1:** Patients with PV during COVID-19 period

	HIV viremia			Number of patients			Total cohort
	Before PV	During COVID-19 period	After PV	Period A (n = 73)	Period B (n = 26)	COVID-19 period (A + B) (n = 99)	COVID-19 period (A + B) (n = 3537)
Group 1	PV	PV	PV	25 (34%)	11 (42%)	36 (36.4%)	36 (1%)
Group 2	NV	PV	PV	34 (46%)	2 (7.6%)	36 (36.4%)	36 (1%)
Group 3	NV	PV	NV	8 (11%)	6 (23%)	14 (14.2%)	14 (0.4%)
Group 4	NV	PV	NA	6 (82%)	7 (27%)	13 (13.2%)	13 (0.4%)

*PV* positive viremia, *NV* negative viremia, *NA* not available

## Discussion

As the effect of SARS-CoV-2 pandemic on HIV continuum of care is still unclear, this study explores whether PV was more frequent during COVID-19 period compared to the previous year.

Here, we show that interventions and efforts adopted by our Infectious Division to mitigate the pandemic impact on care continuum were effective. In fact, the percentage of people with PV during the COVID-19 period was even lower than in the pre-COVID-19 time (2.8% vs 7%), considering a comparable number of patients on stable ART in the two time intervals (3537 vs 3576) and a comparable testing rate (all subjects of the two cohorts were tested at least one for plasmatic HIV viremia during a time window of 9 months). Anyway, other medical Centers reported different experiences, such as Spinelli and colleagues from San Francisco (USA), who observed a higher percentage of unsuppressed HIV patients during pandemic [1]. The Lancet Editoral of November 28, 2020 [10], let us reflect on the opportunities we may derive from this pandemic. In fact, it forced the health system to implement services to reach outpatients without in-person interactions, and that could be a new important resource in the health care system organization, even in future, both in high-resources areas and low-middle income countries.

In our study, a consistent percentage of patients (36.4%), corresponding however to few patients with PV during COVID-19 period, showed a poor virological control even in the previous year, suggesting reasons of virological failure different from the emergency situation, such as poor adherence to ART (group 1). Those patients should be prioritized in the health care process, being a group of people at higher risk of loss at follow up during extraordinary situations that limit access to health facilities [11].

A small number of patients (group 3) had single isolated PV during pandemic and the reasons could be independent of the pandemic.

The group 2 includes probably the patients whose viremia was influenced by the pandemic and represent only the 1% of the entire “COVID-19 cohort”. This group reveled persistent PV in the COVID-19 period, compared to a NV in the pre-COVID-19 time, and it was more numerous in the period A than B, as attended considering the initial unpreparedness of health system and population towards the emergency. These patients could have suffered from both mobility restrictions due to mandatory and strict lockdown and fear to acquire the SARS-CoV-2 infection. Outpatient in-person visits were shifted to remote encounters, when possible, but blood controls and withdrawal of ART were more difficult to track. Delays in ART refills have also occurred, especially during the first 2 months of pandemic (March–April 2020), as previously described [8], and viral load and CD4 monitoring, globally recognized fundamental measures of ART success, may also be delayed or missed during pandemic. Moreover, even after the first period of pandemic, a small proportion of patients preferred not to get in touch with health care facilities, being afraid to acquire the SARS-CoV-2 infection.

Finally, our findings highlight as during pandemic most of our HIV patients maintained viral suppression, even if the number of missed follow visits increased. Indeed, the percentage of people with PV during the COVID-19 period diminished compared to the previous year, confirming the importance to have a flexible health service with a good informatic support and home delivery system. Moreover, we underlined the importance of improving HIV counseling even more during an emergency period, as we done.

The main limitation of our study is that we could not investigate what happened during the intervals between HIV viremia tests in the study periods. Moreover, we considered only patients from our Center and a relatively short period of time. We also didn’t evaluate the impact of this pandemic on other outcomes, as comorbidities, being necessary a longer observation period.

## Conclusion

In term of viral suppression, we successfully managed to ensure continuum of care during pandemic for most of our HIV outpatients throughout telemedicine, and home delivery services for ART, highlighting the importance of a continuous counselling to maintain patients’ adherence to care process.

## Data Availability

The datasets used are available from the authors upon reasonable request.

## Abbreviations

ART: Antiretroviral therapy
ASST: Azienda socio sanitaria territoriale
NA: Not available
NV: Negative viremia
PV: Positive viremia
VL: Viral load
